# Supplementary material for: Comment on Pescott & Jitlal 2020: Failure to account for measurement error undermines their conclusion of a weak impact of nitrogen deposition on plant species richness
Source: PeerJ. 2021 Jan 12;9:e10632. doi: 10.7717/peerj.10632 (PMC7810039; doi:10.7717/peerj.10632)

Supplementary Figure A: Estimated mean of the random spatial field fitted to the data using the SPDE approach in INLA.


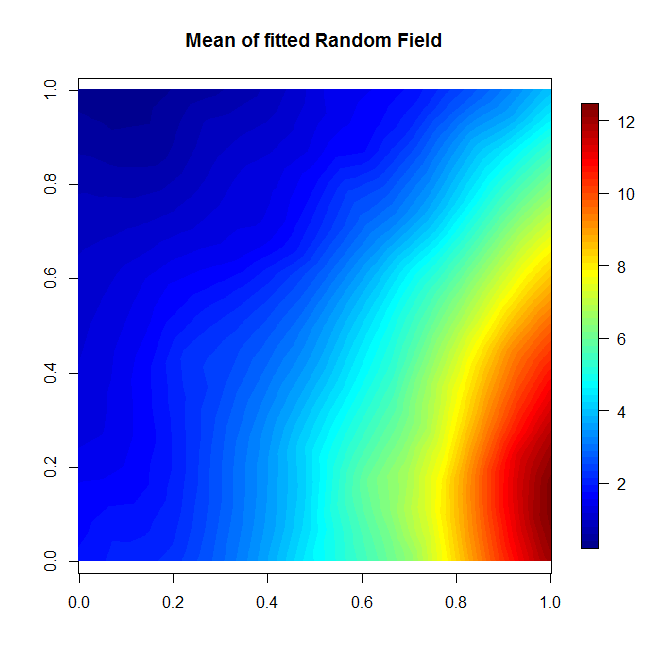

Supplement: Supplemental Information 1 [file peerj-09-10632-s001.docx]
