# Supplementary figures and images for: Comment on Pescott & Jitlal 2020: Failure to account for measurement error undermines their conclusion of a weak impact of nitrogen deposition on plant species richness
Source: PeerJ. 2021 Jan 12;9:e10632. doi: 10.7717/peerj.10632 (PMC7810039; doi:10.7717/peerj.10632)

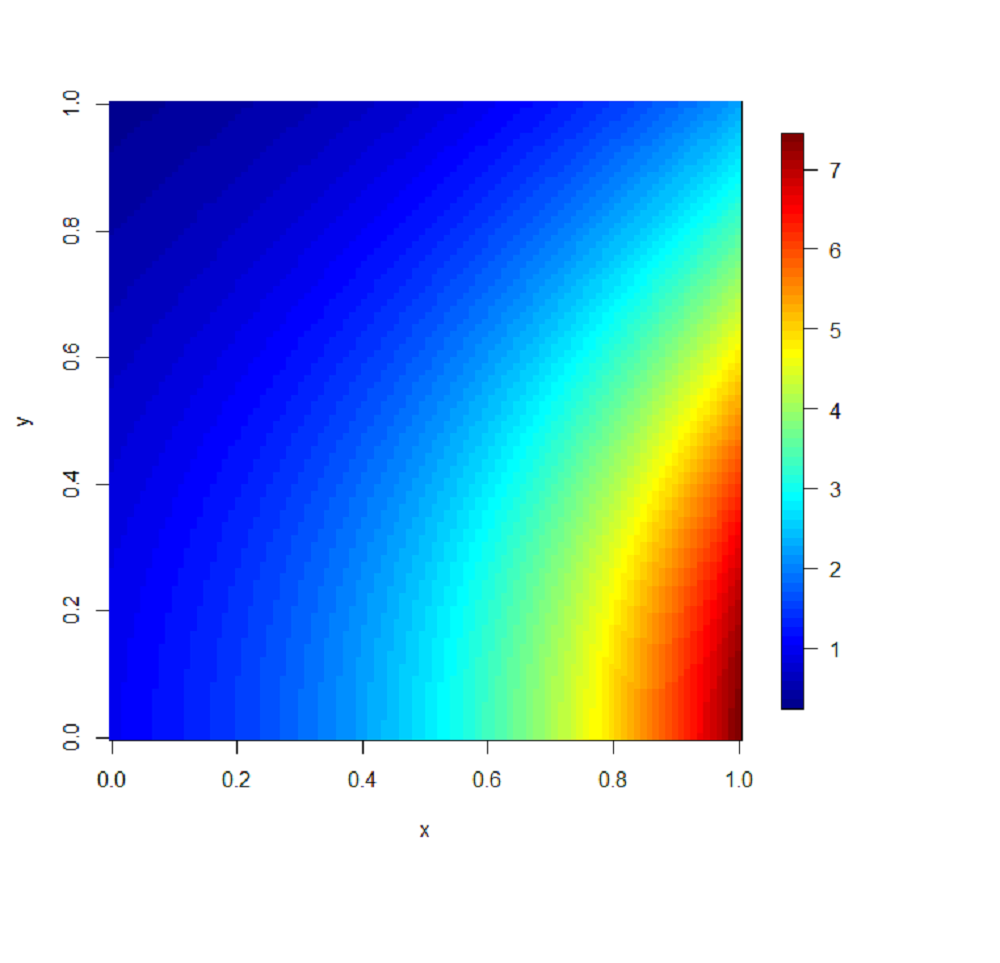

Supplement: Supplemental Information 3 [file peerj-09-10632-s003.png]

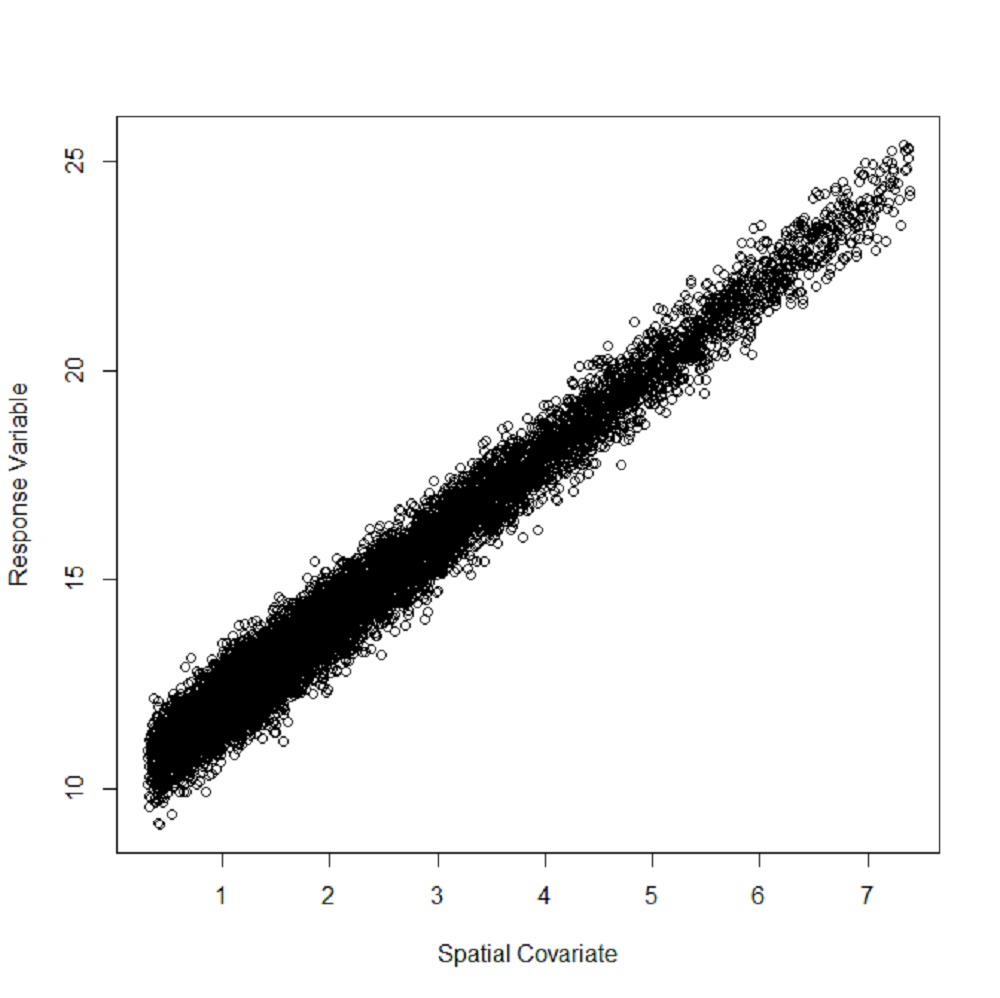

Supplement: Supplemental Information 4 [file peerj-09-10632-s004.png]

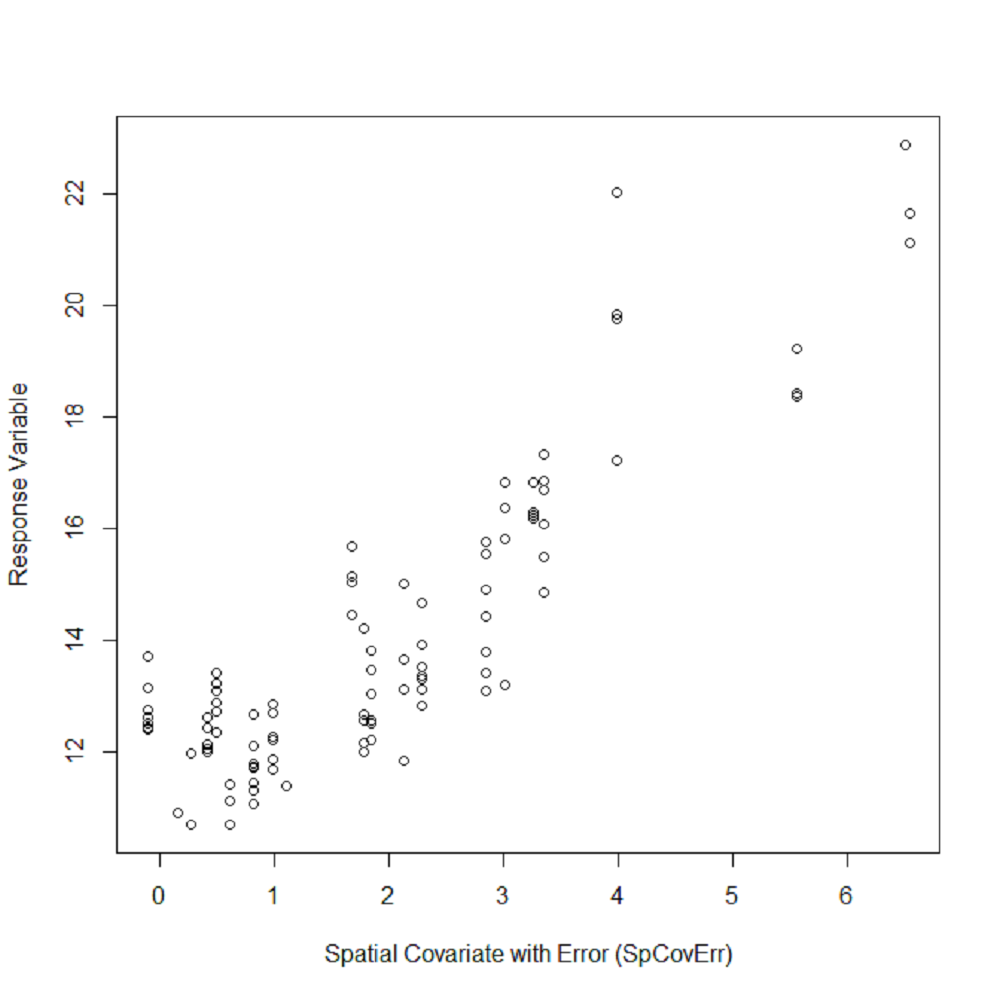

Supplement: Supplemental Information 5 [file peerj-09-10632-s005.png]

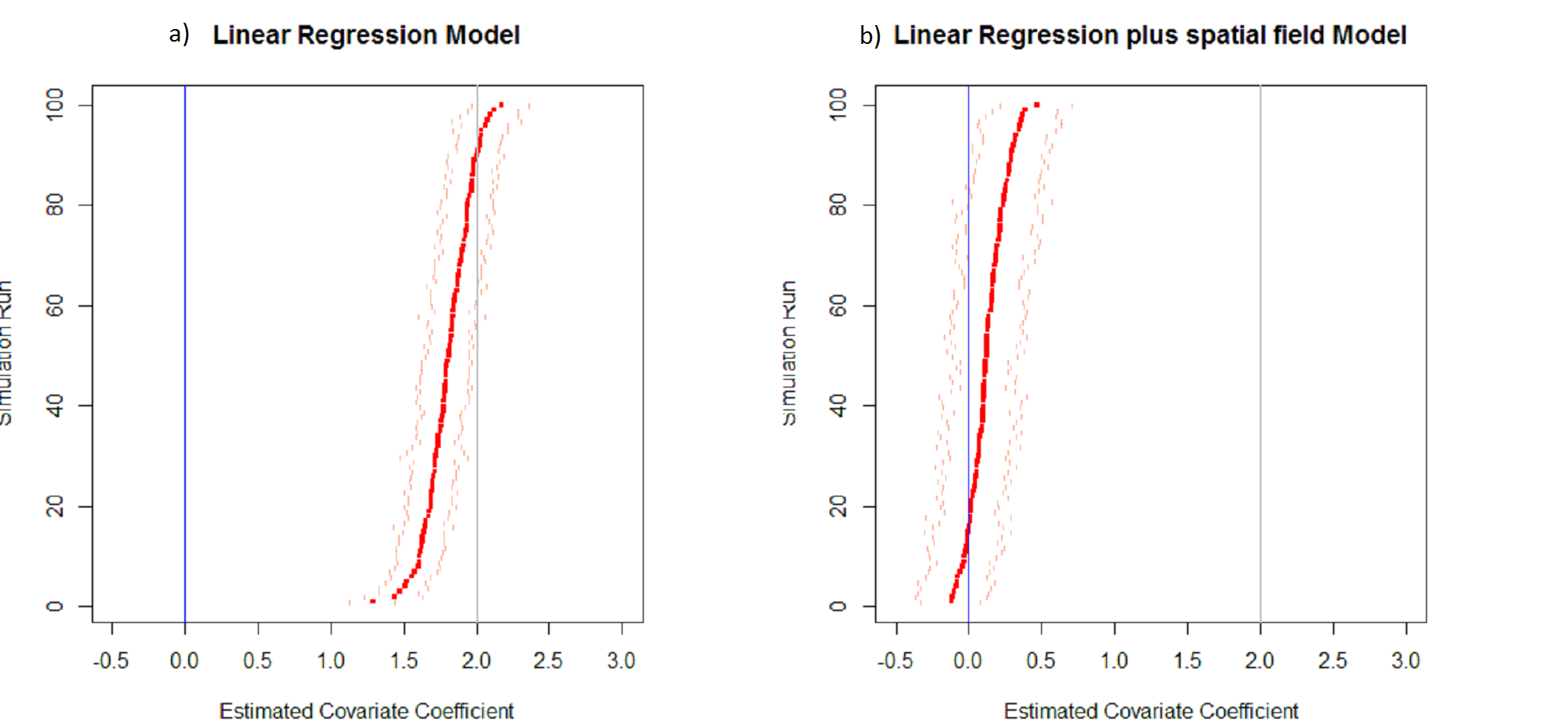

Supplement: Supplemental Information 6 — Parameters derived from a) a simple linear regression model; and b) a linear model plus the addition of a spatial random field. In both plots credible intervals on estimates are shown using tick marks, the value of 0 is included as a blue line and the true value of the coefficient (2) is shown in grey. [file peerj-09-10632-s006.png]

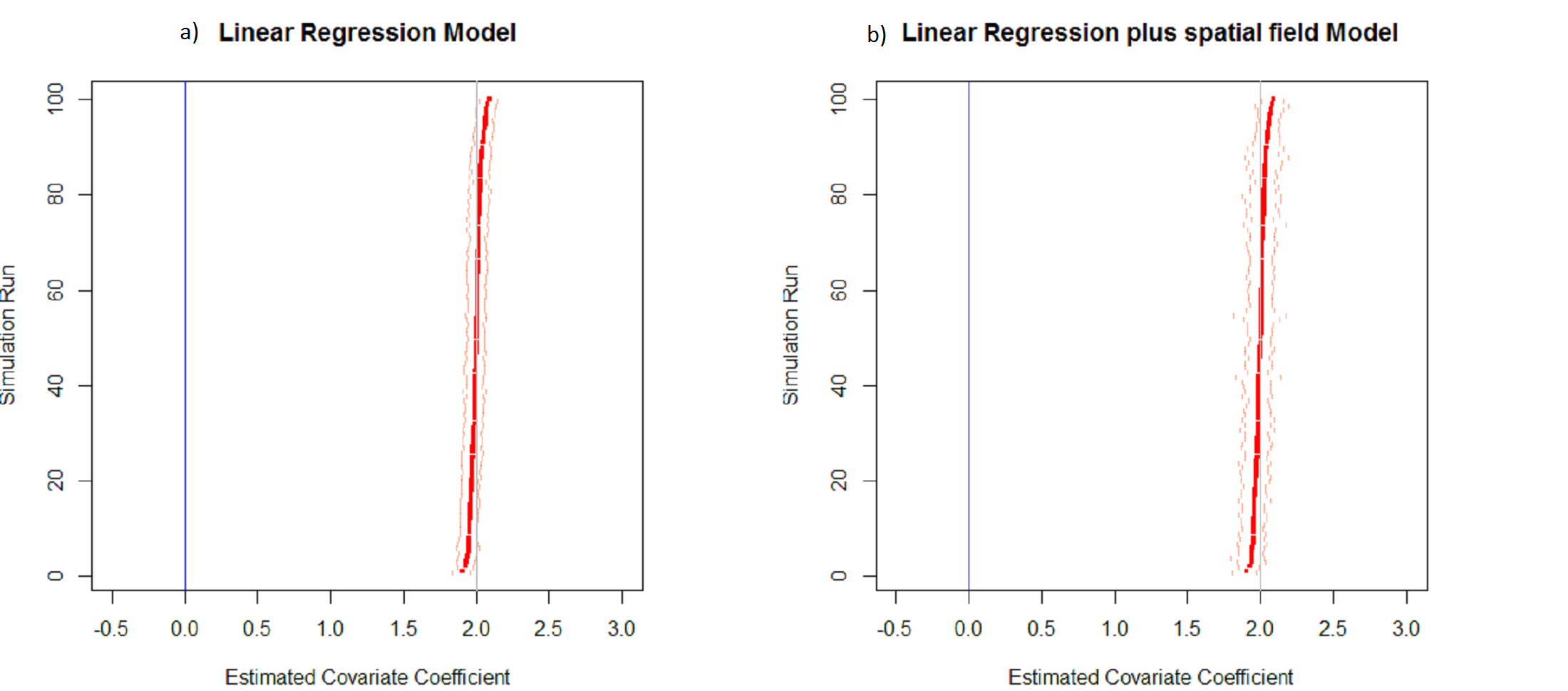

Supplement: Supplemental Information 7 — Parameters derived from a) a simple linear regression model; and b) a linear model plus the addition of a spatial random field. In both plots credible intervals on estimates are shown using tick marks, the value of 0 is included as a blue line and the true value of the coefficient (2) is shown in grey. [file peerj-09-10632-s007.png]

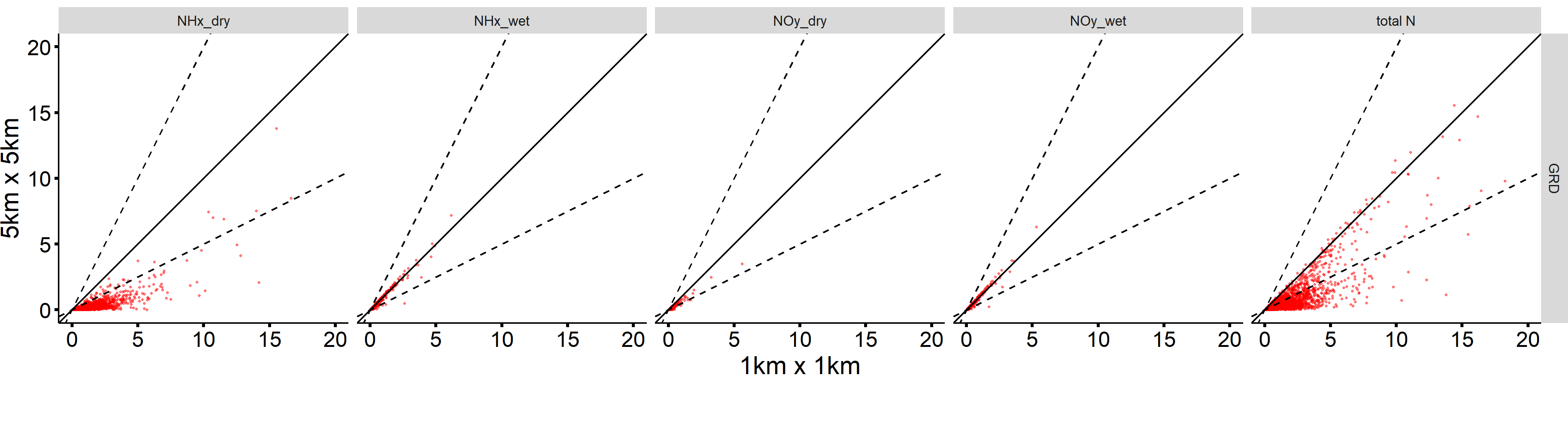

Supplement: Supplemental Information 8 [file peerj-09-10632-s008.png]
